# Using systems thinking to guide the dissemination of the European code against cancer, 5th edition

**DOI:** 10.1002/1878-0261.70195

**Published:** 2026-01-16

**Authors:** Erica D'Souza, David Ritchie, Hajo Zeeb, Joachim Schüz, Carolina Espina

**Affiliations:** ^1^ Environmental and Lifestyle Epidemiology Branch International Agency for Research on Cancer Lyon France; ^2^ Department of Prevention and Evaluation Leibniz‐Institute for Prevention Research and Epidemiology ‐ BIPS GmbH Bremen Germany; ^3^ Health Sciences Bremen University of Bremen Germany

**Keywords:** cancer prevention, dissemination, European Code Against Cancer, knowledge mobilisation, systems thinking

## Abstract

Knowledge mobilisation is essential in cancer prevention to equip individuals with the knowledge to reduce their risk and improve their health. Hence, dissemination of evidence‐based recommendations is a key tenet in the fight to reduce the burden of cancer in the European Union (EU). Systems thinking was used to guide the methods of three substudies involving stakeholders in identifying dissemination actions to enhance the awareness and uptake of the European Code Against Cancer, 5th edition (ECAC5), including mapping barriers and facilitators to achieve impactful dissemination. The proposed actions aimed to foster collaboration and partnership across diverse sectors, utilising diverse and accessible channels to deliver visually engaging content to maximise the delivery and impact of the ECAC5 to the general public in the EU. Many of these actions were evaluated by participants as highly feasible and impactful, thereby supporting their implementation.

## Graphical abstract

Knowledge mobilisation is vital for cancer prevention. Using systems thinking, three substudies engaged stakeholders to identify effective strategies to disseminate the European Code Against Cancer, 5th edition (ECAC5). Stakeholders mapped barriers and facilitators and proposed actions focused on collaboration, accessible communication channels and visually engaging content to enhance ECAC5 awareness and uptake across the general public in the European Union.

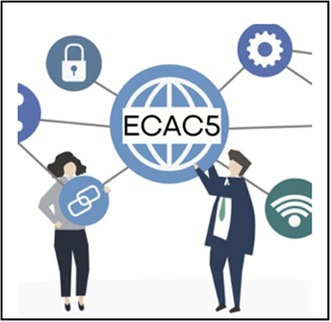

**Abbreviations**
ECAC: European Code Against Cancer
ECAC4: European Code Against Cancer, 4th edition
ECAC5: European Code Against Cancer, 5th edition
ECL: Association of European Cancer Leagues
EU: European Union
NCDs: Noncommunicable diseases

## 1 Introduction

**Fig. 1:**
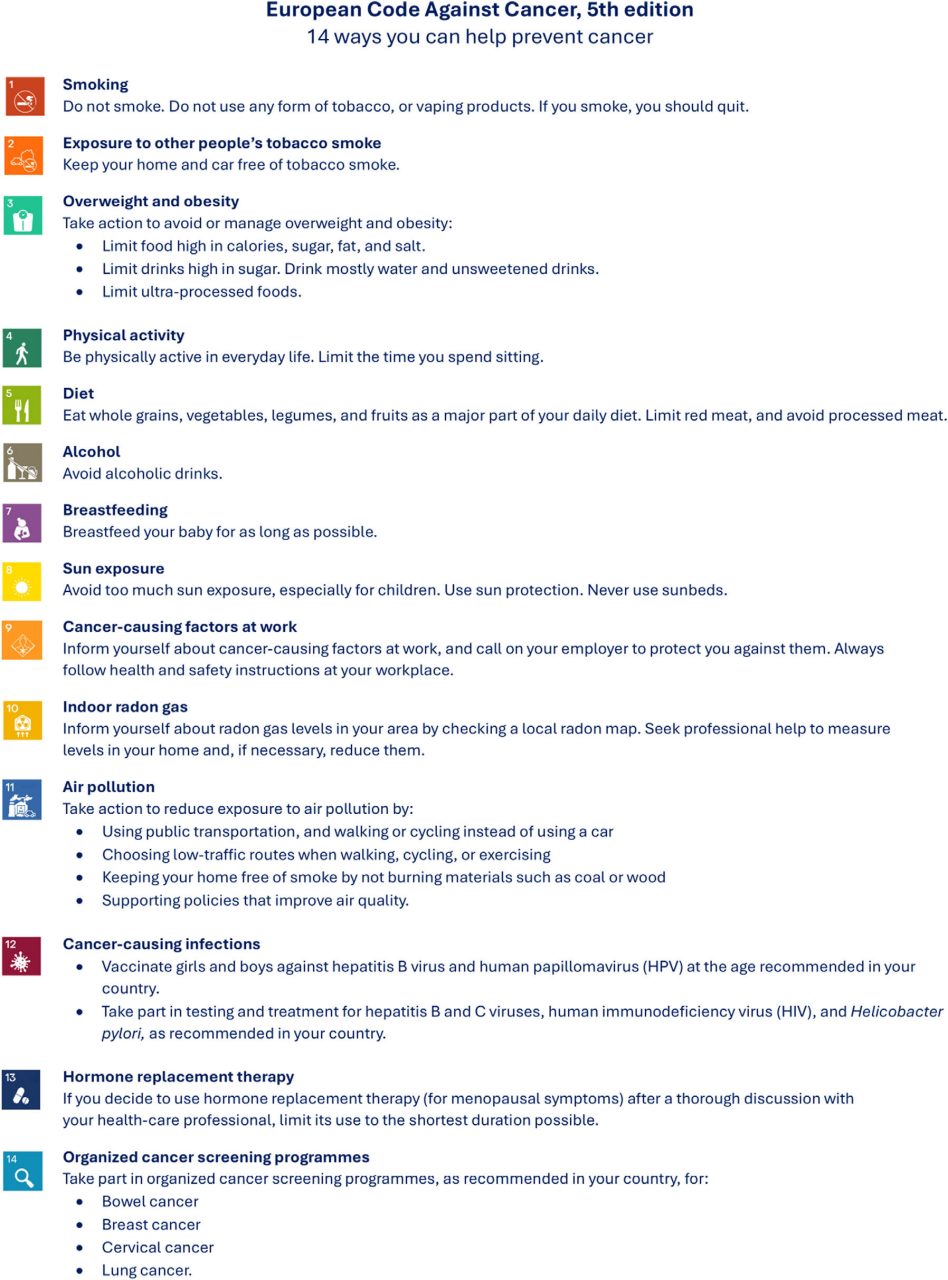
European Code Against Cancer, 5th edition: recommendations for individuals. The 14 recommendations of the European Code Against Cancer, 5th edition (ECAC5) adopted by the Scientific Committee of the ECAC5 project. © 2026 International Agency for Research on Cancer / WHO. Used with permission.

Knowledge mobilisation is an integral part of public health, and in turn cancer prevention, supporting global efforts to reduce population cancer burden. It is defined as the generation and movement of useful and robust knowledge where it can be most impactful [1]. In this vein, the European Code Against Cancer (ECAC) aims to inform the general public in the European Union (EU) about evidence‐based actions to reduce their cancer risk [2]. It is an initiative of the European Commission, which has been disseminated by stakeholders since its inception in the 1980s. Europe's Beating Cancer Plan supports actions to raise the visibility of the ECAC and has set an ambitious goal to make at least 80% of the population aware of it by 2025 [3]. Therefore, novel ways to maximise and expand the reach of the ECAC for its 5th edition (ECAC5) are required [4] (Fig. 1 and Annex S1).

Currently, a number of civil society organisations are contributing to the promotion of the ECAC, albeit with differing levels of involvement. Some examples include dedicating a month of a two‐year calendar to promote one ECAC recommendation, organising campaigns, and using its messages in advocacy. Typically, promotion of the ECAC to the general public is undertaken by these organisations [5]. However, despite sustained promotional efforts, overall public awareness of the ECAC remains low [6, 7, 8]. Across 8171 adults from eight EU Member States, awareness ranged from just 2% in the United Kingdom to 21% in Hungary and Poland [6]. Similarly, among 1520 Swedish adults, only 3.7% recognised the ECAC [7]. The scattered and inconsistent dissemination of ECAC4, combined with limited literature, makes it difficult to measure and evaluate the extent of its reach.

In recent years, more people across the EU are using the internet for health information [9] thereby making this a key dissemination channel. However, this in turn has led to an infodemic, that is the rapid spread of misinformation via social media platforms and other digital outlets creating a significant issue for public health [9]. A 2015 review found that these platforms are unregulated resulting in the opportunity for anyone to deliver health information that is often anecdotal and not backed up with evidence [10]. Moreover, the sheer volume of cancer prevention and other public health messaging is a barrier to the dissemination of ECAC [6]. This increases the need for a highly literate population, including high levels of health and digital literacy. A recent systematic review found low levels of health literacy across the EU, with a third to nearly half of Europeans with low health literacy [11]. Finally, characteristics such as education, gender and socioeconomic status also impact individual engagement with cancer prevention [12] as well as the level of trust in scientists [13].

So far, few studies have applied systems thinking to research dissemination. As systems thinking emphasises cross‐sector collaboration and the co‐production of knowledge [14, 15], stakeholder engagement is needed for effective implementation [16]. Additionally, their varied experiences and insights enhance the relevance and acceptance of dissemination activities while building consensus and working towards a common goal [14, 17]. Civil society organisations, including cancer leagues and nongovernmental organisations (NGOs), are essential in the dissemination of ECAC5 [6]. With strong community connections and established communication networks, they help tailor and deliver cancer prevention messages to diverse audiences, raise awareness and mobilise support. Their involvement ensures that ECAC5's evidence‐based recommendations are accessible and relevant at local, national and regional levels.

For knowledge mobilisation, one such systems thinking approach is the identification of leverage points, wherein a small change in parts of the system can produce a big shift in overall system function [15, 18]. Haynes and colleagues [15] describe six areas of leverage (paradigm, goals, structures and rules, feedback, relationships and power, actors and elements) to target when designing interventions. At the upper levels of the hierarchy, that is, paradigm and goals, change is harder to achieve but is likely to be sustainable and transformational. Knowledge mobilisation research often lacks consideration of broader contexts and fails to account for the complex and dynamic nature of dissemination systems [15]. On the contrary, systems thinking offers a pluralistic view of how processes are influenced by multiple parts of a system, while being adaptive and responsive to changing contexts and needs [15]. This presents an opportunity to harness our efforts to increase the reach of the ECAC5 across the diverse EU general public through stakeholders' engagement as part of the system.

Design thinking methods, like those of systems thinking, stress the importance of context and collaboration with stakeholders to develop sustainable solutions. This is executed through a creative and iterative problem‐solving process that encompasses empathising, ideating, testing, learning and refining thereby ensuring that solutions are valuable to the needs of its beneficiaries [16, 19]. It has been found to be especially effective for actions that engage young people, when they play a purposeful role in matters affecting them [20, 21]. Design thinking stems from the need to apply fresh approaches when tackling complex problems [16, 22], a crucial element to aid in the identification of novel and unique approaches to disseminate ECAC5 to younger populations. It is increasingly being used in public health and healthcare interventions [23, 24], including those for cancer control [16, 17, 25, 26].

**Fig. 2:**
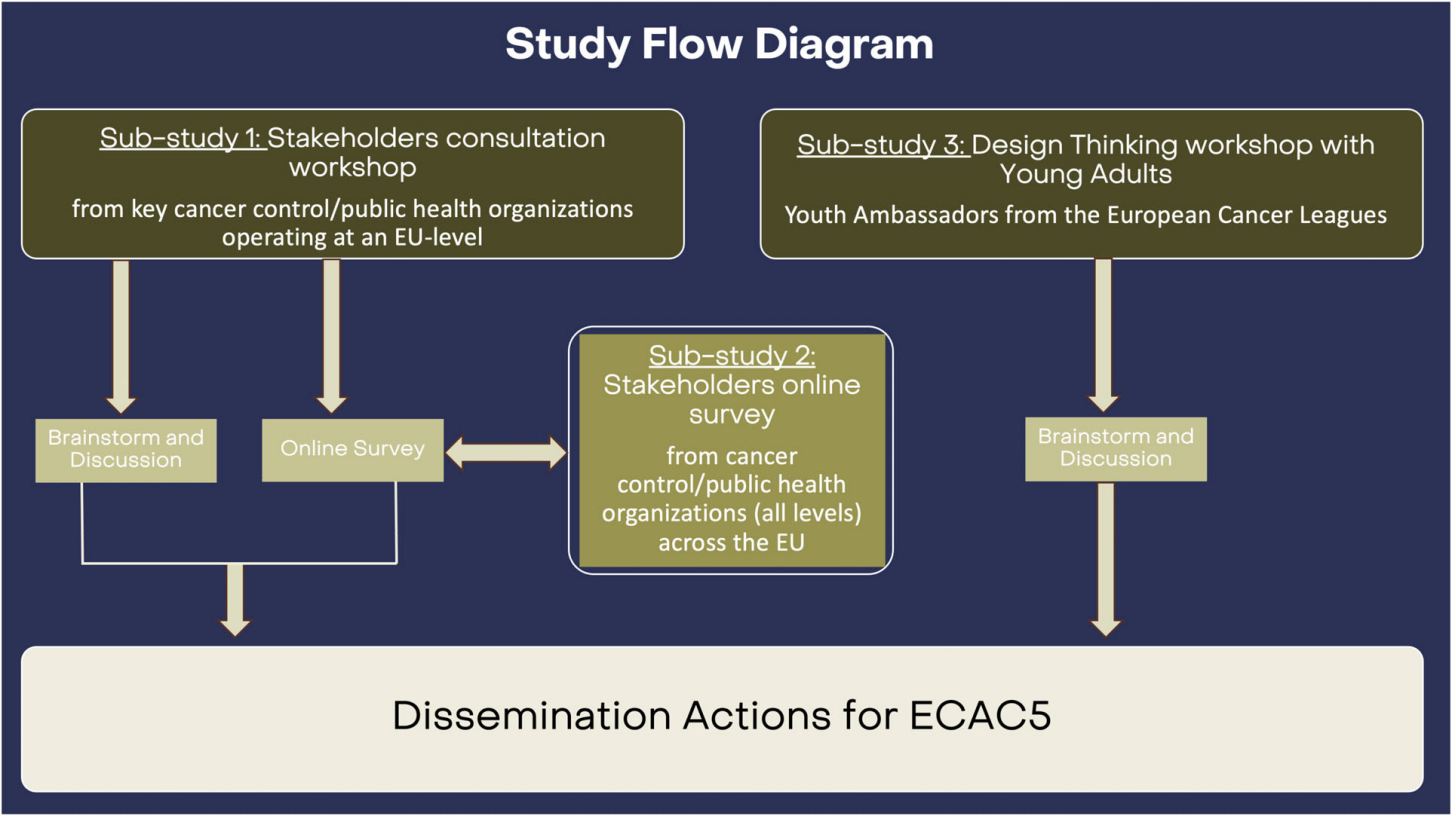
Study flow diagram. The figure shows the activities undertaken in each substudy and the relationships between them to achieve the overall aim of improving the dissemination of ECAC5.

In the context of evolving public health challenges, the dissemination of ECAC5 demands innovative, systems‐informed and participatory approaches. This article presents three empirical substudies that were designed to identify valuable strategies to enhance the dissemination of the ECAC5 (Fig. 2). By applying principles from systems thinking and design thinking, this work underscores the critical role of stakeholder engagement—particularly that of civil society organisations and young people—in the cocreation and contextualisation of knowledge mobilisation efforts.

## 2 Materials and methods

The overall aim was to learn from EU stakeholders involved in knowledge dissemination and health promotion in cancer control/public health and identify strategies applicable across diverse EU contexts to enhance the dissemination of the ECAC5. Additionally, a secondary outcome was to foster collaboration and partnership among stakeholders to develop sustainable dissemination across the EU. This has been achieved in an ECAC5 Partnership Declaration signed by a large number of stakeholders (31 as of 1 December 2025), committed to promote education and awareness‐raising initiatives at EU‐level and locally, to encourage the uptake of ECAC5 recommendations [27]. To achieve this, three substudies were conducted (Fig. 2).

### 2.1 Substudy 1: stakeholders consultation workshop

The aim was to consult key stakeholders to identify, evaluate and prioritise dissemination strategies for ECAC5 based on the current dissemination landscape in the EU. The target population was stakeholders from key cancer control and public health organisations who were identified through different networks and invited to propose dissemination actions for ECAC5. Purposeful sampling was used to identify potential participants through the research teams' professional networks. The two‐hour workshop was conducted online via *MS Teams* held in February 2024, facilitated by three members from the research team.

Out of the 19 organisations invited via email, 13 participants from 11 organisations across the EU agreed to participate and attended the workshop. Participants belonged primarily to EU‐level cancer prevention and control organisations (*n* = 7), public health associations (*n* = 3) and one national cancer league (*n* = 1). Participants ranged from directors to policy officers, health promoters and communications experts.

Thirteen questions and eighteen subquestions on knowledge mobilisation strategies using a systems perspective developed by Irving and colleagues [28] were emailed to participants prior to workshop commencement as a primer to encourage reflection on possible systems‐level strategies for dissemination of ECAC5. This fed into divergent (individual brainstorm) and convergent (group discussion, evaluation and consensus) activities that enabled participants to discuss the current EU dissemination landscape and identify dissemination actions for ECAC5. This list of actions was noted by the research team during the workshop to be used as content for a follow‐up online survey.

Postworkshop, participants received a link to an online survey (Annex S2) with a list of their proposed dissemination actions to evaluate and prioritise for feasibility and impact on a five‐point Likert scale based on their professional expertise and work in the field. Feasibility was defined as an ‘action being easy to implement’, while impact was defined as ‘the ability to reach the EU general population and in turn improve cancer prevention’. Participants scored each action from 1 to 5, with the latter indicating high levels of feasibility and/or impact. This reflective exercise was used to encourage participant evaluation of the actions proposed and indicate, via a score, which are most recommended for ECAC5.

The six leverage points for changing complex systems (paradigm, goals, structures and rules, feedback, relationships and power, actors and elements) [15] were used to categorise the actions identified for the dissemination of ECAC5.

### 2.2 Substudy 2: stakeholders online survey

As an extension of substudy 1, substudy 2 engaged further with other relevant EU stakeholders with a broader scope in their mission via an anonymous online survey hosted on *MS Forms*. Reusing the survey from substudy 1, substudy 2 aimed to identify barriers and facilitators to disseminating in the EU and provide additional evaluation and prioritisation of the dissemination actions identified in substudy 1. The target population was stakeholders including public health, noncommunicable diseases (NCDs), and cancer prevention and control organisations identified through EU networks. An email invitation with the survey link was sent to potential participants, identified via purposeful sampling within the research teams' networks. Additionally, to increase the response rate, a snowball sampling approach was employed, as invitees were requested to forward the survey invitation email to those deemed suitable within their networks. Given the descriptive intention of this substudy, no sample size and power calculations were performed.

Eighteen responses were received. Participants were primarily communication or project officers (*n* = 7), followed by managers or leaders without classification specified (*n* = 4), executive director or president (*n* = 2), public affairs lead (*n* = 1), specialist (*n* = 1), policy‐makers (*n* = 1), researchers (*n* = 1), and technical secretariat (*n* = 1). Participants came mainly from civil society/nongovernmental organisations (*n* = 7), followed by organisations dedicated to cancer prevention and treatment (*n* = 4), advocacy (*n* = 2) and research (*n* = 2). There was also one representative each from an international organisation, communications agency and government hospital.

The online survey utilised in substudy 1 (Annex S2) was repurposed to evaluate and prioritise the identified dissemination actions for feasibility and impact, based on participants' professional expertise and work in the field. Additionally, information on barriers and facilitators when disseminating health‐related information to the EU general public was gathered via two open‐ended questions. The study methodologies were approved by the IARC Ethics Committee (IEC 24‐16‐A1).

For the analysis, seventeen dissemination determinants identified by Baumann and colleagues [29] were used to theme and categorise dissemination barriers and facilitators. As in substudy 1, the average score for feasibility and impact on each action was calculated to evaluate the feasibility and impact of implementing the proposed dissemination actions.

### 2.3 Substudy 3: youth design thinking workshop

Using design thinking, this substudy aimed to explore young adults' perspectives on how to improve the dissemination of the ECAC5 to younger population groups within the EU. The target population for this workshop was current members (*n* = 30) of the European Cancer Leagues' Youth Ambassadors programme (purposeful sampling), chosen for their motivation and involvement with previous editions of the ECAC [30]. These Youth Ambassadors empower young adults (18–35 years) by spreading ECAC messages and advocating for cancer prevention across the EU. All communication with participants was conducted through the two programme co‐leaders, who also promoted, recruited and invited potential participants to the workshop. The setting was an online, half‐day workshop hosted on *Zoom*. Four ECL staff supported the two facilitators from the research team. The workshop was attended by eighteen youth ambassadors from across the EU.

**Fig. 3:**
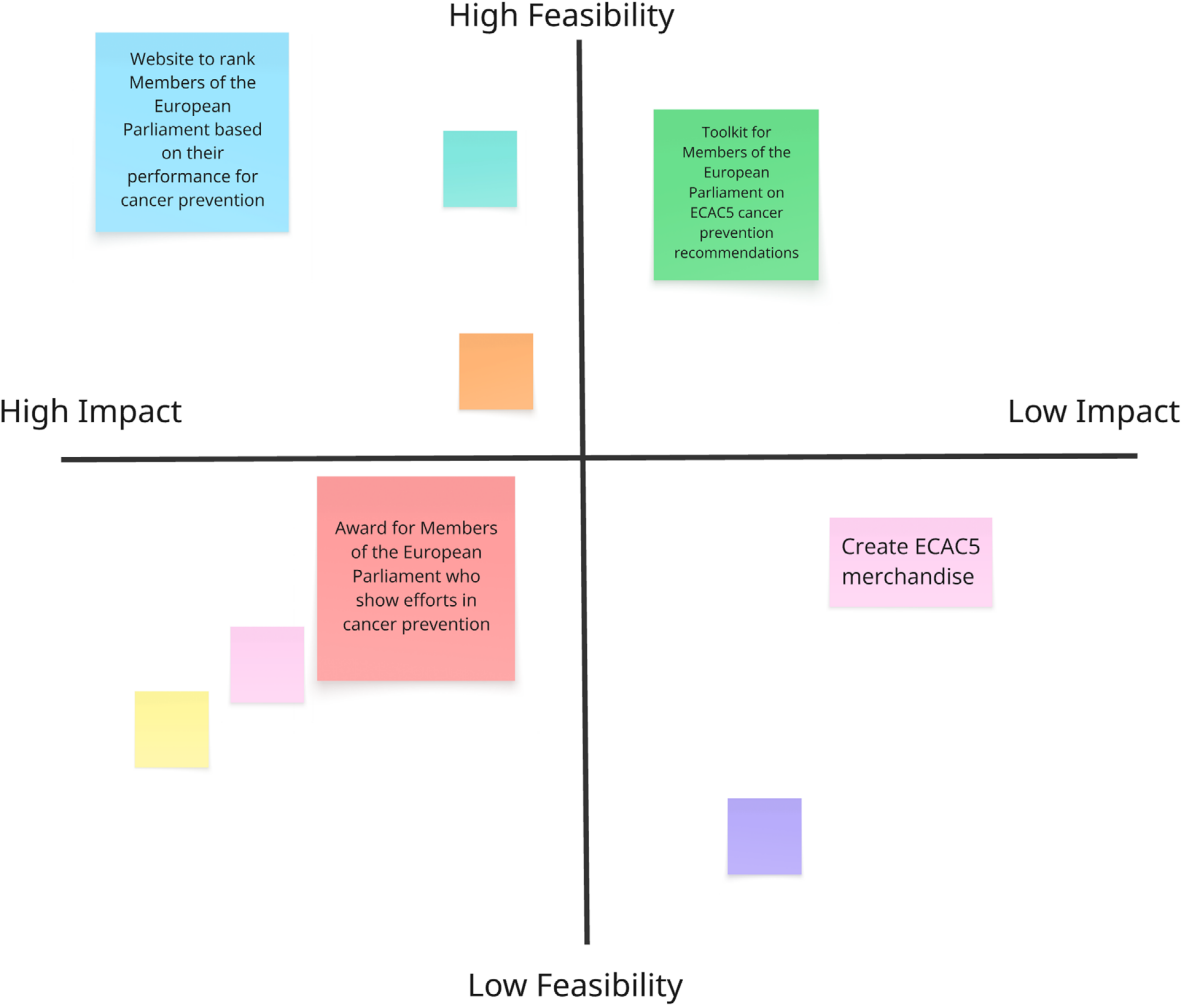
Illustrative representation of the Feasibility and Impact Evaluation Matrix used in substudy 3 to evaluate brainstormed dissemination actions. The matrix shows the degree of the potential feasibility and impact of each brainstormed dissemination action as reflected upon by substudy 3 participants. For illustrative purposes, only four examples have been highlighted. The blank boxes exemplify other brainstormed ideas that were evaluated by this group.

Prior to the workshop, participants were provided with a short overview on the five‐step Design Thinking process and requested to complete an activity in preparation for the workshop. Participants were divided across four groups and completed the following activities in relation to the five‐step process:

- **Empathy mapping** (preworkshop): Each participant conducted an interview one young adult to elicit their perspective on the barriers and facilitators to receiving health information, and in particular cancer prevention information. Participants were encouraged to interview their peers or those known in their networks. Some examples of the semi‐structured interview questions included:
- Channels and sources of health information commonly accessed.
- Examples of memorable health information campaigns.
- Challenges faced when accessing health information.
- Improving the dissemination of health information.
- **Define** (preworkshop): Typically, this step occurs during the workshop. However, we defined the problem question, ‘How to improve the dissemination of ECAC5 to young adults in the EU?’ prior to the workshop to ensure we meet the objectives of our overall aim.
- **Ideate** (during workshop): Divergent (individual brainstorm) and convergent (group discussion and brainstorm) activities to identify possible dissemination strategies were conducted. This was followed by a consensus building activity (Fig. 3), where each group evaluated the feasibility and impact of brainstormed strategies to lead them to an agreement on one idea to prototype and test.
- **Prototype** (during workshop): To adapt to the online structure, in their respective groups, participants developed a detailed storyboard of their design solution (chosen dissemination strategy) to reflect on and share (via powerpoint) with the other groups for feedback. This feedback is used to refine the design solution before being tested.
- **Test** (postworkshop): After the workshop, as part of their role as an ECL youth ambassador, each group was encouraged to pilot test and refine their solution using the current edition of the ECAC to inform dissemination activities for the future dissemination of ECAC5.

As in substudy 2, Baumann et al.'s [29] dissemination determinants were used to theme and categorise the barriers and facilitators identified when disseminating to young adults in the EU. Like substudy 1, Haynes et al.'s [15] six leverage points were used to categorise the dissemination strategies.

## 3 Results

The results of the three substudies are described below, each contributing to the overarching aim of identifying dissemination strategies for ECAC5. This section begins with an overview of the barriers and facilitators when disseminating to the EU general public—a useful antecedent to identifying dissemination actions. However, as such discussions took place anecdotally during substudy 1, only data from substudies 2 and 3 are presented (Table 1). Next, the dissemination actions proposed by participants in substudies 1 and 3 are described and categorised (Table 2). Dissemination actions proposed in substudy 1 were evaluated for feasibility and impact by participants in substudies 1 and 2.

**Table 1:** Barriers and facilitators when disseminating to the EU general public.

Dissemination determinants^a^ [29]	Barriers	Facilitators
**Source of knowledge**	Concerns about the lack of authority among current disseminators and the absence of a central, credible information hub (substudy 2)	Use of trusted/authoritative messengers—such as healthcare professionals, government platforms and recognised influencers—was identified as an effective strategy to enhance dissemination (substudy 2)
**Medium of communication**	Difficulties identifying appropriate platforms, and an over‐reliance on free or fragmented methods (substudy 2); and lack of trust in social media, which was often seen as prioritising sensationalism over accuracy (substudy 3)	Using a wide range of accessible channels (substudy 2); and the creation of a centralised and trustworthy ECAC5 platform would support dissemination efforts (substudy 3)
**Content of communication**	Generalised messaging, excessive jargon, stigmatising language and unbalanced focus on certain risk factors (substudies 2 and 3); and conflicting or misleading information, especially via social media, contributed to confusion (substudy 3)	Strengths‐based and culturally sensitive messaging, the use of lived experiences, visuals, and interactive design, as well as clear, concise, and actionable guidance (substudies 2 and 3); and avoiding oversimplification while maintaining accessibility and relevance (substudy 3)
**Audience**	Identifying the appropriate target audience (substudy 2)	
**Complexity of innovation**	Information overload can lead to feelings of overwhelm and demotivation, reducing engagement with ECAC5 content (substudy 2)	
**Triability of innovation**		Implementation of multilevel and multisectoral dissemination strategies as key to improving reach and access (substudy 2)
**Observability of the results**		Dissemination could be strengthened by regular evaluation to inform future actions and improvements, and publication of dissemination efforts and stories of success are also key facilitators (substudy 2)
**Salience of information**	Lack of perceived salience, particularly among young people and those without direct cancer experiences, and cancer's perceived distance into the future made it a low‐priority concern, especially for younger groups (substudies 2 and 3)	
**Users' perceived attitude towards the innovation**	Cancer prevention information can elicit fear, overwhelm or disengagement, and a broader mistrust of scientific and cancer prevention information, particularly in the aftermath of the COVID‐19 pandemic (substudy 2)	
**Compatibility of the innovation with the setting**	ECAC5's lifestyle recommendations may be unachievable for some groups, such as individuals from lower socioeconomic backgrounds (substudy 2)	Targeted communication—tailored to different groups/sectors of the general population (substudies 2 and 3); adapting messaging to audience‐specific needs and preferences was viewed as critical to increasing both reach and impact (substudies 2 and 3); and translating ECAC5 into multiple EU languages (substudy 2)
**Context**	Difficulties in making ECAC5 messages stand out amidst the vast array of competing information (‘noise’), and cultural factors—including cancer‐related stigma in certain communities—further impeded dissemination (substudy 2)	Presence of policy support for dissemination activities community‐led initiatives, and promoting ECAC5 in schools (study 2)
**Interpersonal networks**	Lack of coordination and engagement among key stakeholders (substudy 2)	Influence of ‘Multipliers’—individuals who share within their communities thereby multiplying the message (substudy 2)
**Opinion leaders and change agents**		Potential of leveraging celebrities, public figures, and partnerships with aligned organisations (i.e. those that share the same goals) as key to facilitating health information (substudy 2)
**Capacity**	Low health and digital literacy in some groups of the general public cause disengagement, as most information was disseminated digitally these days (substudy 2)	

a Baumann et al. [29] identified 17 constructs but only the ones relevant to our study (*n* = 14) are listed in this table.

**Table 2:** Assessment of proposed actions to disseminate ECAC5^a^.

Systems lever [7]	Study of origin	Dissemination actions	Average feasibility score (range 1–5)	Average impact score (range 1–5)	Combined score (feasibility + impact; out of 10)
**Structures and Rules**	1	Push for the use of ECAC5 at policy level and align with national plans in place by engaging in policy dialogues to put cancer prevention on the agenda	3.7	4.3	8
	1	Advocate for incorporating the implementation of ECAC5 at national‐level electoral manifestos	3.2	3.3	6.5
	3	Create a website to rank Members of Parliament on how they have performed for cancer prevention in their election manifesto using a scorecard/barometer	N/A	N/A	N/A
**Feedback & relationships and power**	1	Cocreate ECAC5 dissemination actions and their implementation with key stakeholders	4.1	4.2	8.3
	1	Synergise with other projects at EU level with common values (equity, human rights, etc.)	4.2	4	8.2
	1	Garner support within each country by forming alliances with national‐level patient, scientific and medical associations for dissemination and advocacy with decision‐makers	3.9	4	7.9
	1	Join forces and partner with other European projects/initiatives with common values (e.g. equity, human rights) to amplify ECAC5 messages—making use of their existing audience	4	3.8	7.8
	1	Facilitate coalitions of like‐minded organisations to promote at the ECAC5 at political level nationally	3.9	3.9	7.8
	1	Host an annual EU‐level ECAC forum to show examples of promotion, describe impact and provide the opportunity to showcase and discuss experiences of utilising and implementing ECAC5	3.8	3.5	7.3
	1	Partner with nontraditional stakeholders, and private or public organisations outside of cancer prevention and public health (e.g. banks, telecoms, insurance companies, sports organisations). Take care to ensure no conflict of interests (i.e. commercial determinants of health)	3.5	3.5	7
	1	Partner with insurance companies for reductions/discounts for undertaking positive lifestyle changes based on ECAC5 recommendations (study 3)	N/A	N/A	N/A
	3	Award for Members of Parliament who show efforts in cancer prevention	N/A	N/A	N/A
	3	Collaborate with stakeholders (e.g. tourism) and events (e.g. music festivals)	N/A	N/A	N/A
	3	Collaborate with stakeholders that have the same mission (e.g. sunscreen industry)	N/A	N/A	N/A
**Actors and elements of the systems, including practices and resources**	1	Make ECAC5 available in an online version with clear and simple graphics that can be printed and easily translated	4.7	4	8.7
	1	Create an ECAC5 communication toolkit (i.e. press packs), including press releases for mass media.	4.7	4	8.7
	1	Leverage scientific experts involved in ECAC5 for interviews to explain more about the evidence behind the recommendations of the European Code	4.8	3.9	8.7
	1	Provide a toolkit (best practice examples) of dissemination strategies that are translatable across a variety of contexts (e.g. applicable to various countries in the EU)	4.6	4	8.6
	1	Work with educators and teachers to promote the ECAC5 to children and adolescents (e.g. in educational settings such as schools, youth centres)	4	4.5	8.5
	1	Provide tailored information to explain ECAC5 to policymakers (e.g. policy briefs)	4.4	4	8.4
	1	Purchase targeted advertisements on social media so ECAC5 can reach a wide but targeted cross‐section of the public	4.2	4.1	8.3
	1	Promote ECAC5 in physical formats in high visibility settings (e.g. family doctor/general practice and hospital waiting rooms, pharmacies)	4.4	3.9	8.3
	1	Use social media platforms effectively and create content on each recommendation of the ECAC5 (e.g. Instagram reels—short videos that hold the audience's attention)	4.4	3.9	8.3
	1	Create strengths‐based and positive promotion of the ECAC5 (e.g. with humour) to increase motivation to engage with it, while highlighting how it can be a tool (solution) to prevent cancer	4.2	4	8.2
	1	Identify and use creative and varied channels of dissemination (e.g. podcast interviews with patient representatives and advocates; youtube channel on cancer prevention topics; storytelling through films; cartoons; short videos)	4.2	4	8.2
	1	Learn from comparable and successful dissemination campaigns to extrapolate best practices (e.g. campaigns on HIV prevention and HPV vaccination)	4.1	4	8.1
	1	Create targeted dissemination formats and channels to subgroups in the general public (e.g. by age, gender, vulnerable or at‐risk populations) to improve engagement with ECAC5	4	4	8
	1	Engage public figures and celebrities (e.g. football players, national champions who were patients themselves) to consistently promote the message of cancer prevention and the ECAC5	3.7	4.2	7.9
	1	Engage with trusted social media influencers by demographic or region/country	3.7	3.9	7.6
	1	Engage children and youth in competitions and gamification to promote ECAC5	3.7	3.5	7.2
	3	Work with schools and teachers to embed the ECAC5 in the teaching curriculum	N/A	N/A	N/A
	3	Collaborate with influencers and celebrities to share ECAC5 via their social media	N/A	N/A	N/A
	3	One‐off events (e.g. Health Day in school with a stand for ECAC5 promotion; walks/runs; expositions and workshops at universities)	N/A	N/A	N/A
	3	Street art and billboards on each ECAC5 recommendation, including posters at bus stops	N/A	N/A	N/A
	3	Create ECAC5 merchandise (e.g. an ECAC5 calendar with one recommendation per month, magnets, cookbook)	N/A	N/A	N/A
	3	Advertise ECAC5 on social media	N/A	N/A	N/A
	3	Use humour to create short videos on each ECAC5 recommendation (e.g. use current social media trends, such as memes, to create ECAC5 content)	N/A	N/A	N/A
	3	Digital art (e.g. on each recommendation), gamification (e.g. game of right choices) and competitions (e.g. online quiz) with the ECAC5	N/A	N/A	N/A
	3	Create a toolkit for Members of Parliament on cancer prevention topics	N/A	N/A	N/A
	3	Varied channels of dissemination—youtube, social media campaigns, podcasts	N/A	N/A	N/A

a The table shows the list of dissemination actions proposed in substudies 1 and 3, actions from substudy 1 were further scored by stakeholders from substudies 1 and 2.

### 3.1 Barriers and facilitators to disseminating cancer and health prevention information, including the ECAC, to the EU general public

Several dissemination determinants were identified as either barriers and/or facilitators when disseminating cancer prevention and health information to the EU general public, including young adults (Table 1). The content, that is the message and the information being transmitted, is not only the main barrier but also the main facilitator to improving the dissemination of ECAC5. Ensuring simple, strengths‐based communication, supported by engaging visuals and design, is favourable. Conversely, high levels of technical language and jargon are detrimental to dissemination. Likewise, the context in which dissemination takes place can hinder or support dissemination efforts. For example, in some communities there is stigma attached to cancer creating an obstacle, while on the other hand community‐led initiatives can fortify dissemination efforts. Other barriers include the audience's capacity to engage with the information as it is affected by the level of health and digital literacy.

The medium through which information is presented can also impede dissemination due to the inability to choose the best mode within funding constraints as well as asynchronous dispersion across various channels. Simultaneously, ensuring the channels of dissemination are current, accessible and relevant enables dissemination success. Audiences' attitude towards the innovation (i.e. the ECAC) and their salience of its contents can also affect dissemination uptake. Utilising celebrities or people of influence with a positive image in the general public can promote reach and impact, alongside collaborating with organisations that share the same goals and vision.

### 3.2 Proposed actions to disseminate ECAC5

Table 2 outlines the dissemination actions elicited from brainstorming sessions at the workshops (substudies 1 and 3), categorised according to Haynes et al.'s [15] hierarchy of leverage. As there were no actions proposed at the ‘paradigm’ and ‘goals’ levels, these are excluded from the table. Most actions were at the lowest level: ‘Actors and elements’, with a large focus on the content of communication and various ways (i.e. knowledge brokers, channels) through which ECAC5 can reach the general public. Further up the leverage hierarchy, at the ‘Feedback’ and ‘Relationships and power’ level, most dissemination interventions focused on collaboration and alliances with a variety of partners. Only three dissemination actions were proposed at the ‘Structures and rules’ level, mainly aimed at motivating members of parliament and putting ECAC5 on the policy agenda.

### 3.3 Evaluation of proposed actions to disseminate ECAC5

Feasibility and impact scores, as defined in the Methods and Materials section, were gathered to identify the most recommended dissemination actions proposed by substudy 1 stakeholders. The average feasibility and impact scores for each proposed action were calculated from the survey responses in substudies 1 and 2 (Table 2). These scores are to be interpreted as an indicator of the value of each action based on participants' professional expertise and work in the field of cancer prevention and/or public health.

According to participants, the two most promising strategies (feasibility 4.7; impact 4) were: *Make ECAC5 available in an online version with clear and simple graphics that can be printed and easily translated* and *Create an ECAC5 communication toolkit (i.e. press packs), including press releases for mass media*. Although *Leverage scientific experts involved in ECAC5 for interviews to explain more about the evidence behind the recommendations of the European Code* received the highest score for feasibility (4.8), it only received a 3.9 score for impact. On the other hand, *Work with educators and teachers to promote the ECAC5 to children and adolescents (e.g. in educational settings such as schools, youth centres)* was identified as having the highest impact (4.5), but it was less feasible (4). Finally, *Advocate for incorporating the implementation of ECAC5 at national‐level electoral manifestos* would be the least feasible and have the least impact, closely followed by *Partner with nontraditional stakeholders, and private or public organisations outside of cancer prevention and public health (e.g. banks, telecoms, insurance companies, sports organisations). Take care to ensure no conflict of interests (i.e. commercial determinants of health)*.

### 3.4 Pilot testing proposed actions to disseminate ECAC5 to EU young adults

As part of the design thinking process, substudy 3 decided to pilot test some of their proposed dissemination strategies. Unlike substudies 1 and 2, each of the four groups in the design thinking workshop (substudy 3) used the feasibility and impact task as a sorting activity (Fig. 3) to evaluate and reach consensus on one action to prototype (step 4 of the design thinking process) and test (step 5 of the design thinking process) in their chosen setting. The opportunity to test their selected action was part of their work to fulfil the requirements of the ECL Youth Ambassadors programme and was outside the aims and scope of this project.

The resulting four dissemination projects to be tested (step 5) in the future are as follows:

- A one‐day hybrid health festival (in‐person and online to increase accessibility);
- A toolkit for teachers to embed ECAC in their teaching curriculum, including a focus on one recommendation per month;
- A week‐long online campaign promoting the ECAC;
- A website to increase transparency of cancer prevention and control‐related activities of Members of Parliament, while simultaneously using this as a tool to increase pressure on their commitment to the cause.

## 4 Discussion

The three substudies have proposed 40 examples of possible dissemination strategies that participants deemed feasible and impactful, based on their knowledge and expertise, to increase the reach of the ECAC5 among the EU general public. The breadth of these 40 actions provides EU countries with the flexibility to adopt those most appropriate to their specific populations and contexts. The proposed actions focus on improving the communication of ECAC5, increasing the accessibility and diversity of dissemination channels and collaborating and partnering with those that fall within and outside the traditional scope of public health disseminators. Moreover, to have a population‐wide impact, a few actions were aimed at initiating change at the policy level. The suggested actions directly addressed the barriers identified in the current dissemination landscape. There was a high degree of similarity in the themes of the dissemination actions proposed by participants in substudies 1 and 3. Suggested actions were considered highly feasible and impactful, thereby warranting their implementation to increase the reach of ECAC5.

Collaborative efforts with stakeholders, especially knowledge brokers that understand local contexts, can facilitate knowledge into practice [15]. Hence, their inclusion in exploring and enhancing the dissemination of the ECAC5 was a key tenet of all three substudies. Empowering younger populations with cancer prevention information can encourage the earlier onset of forming favourable lifestyle actions to promote good health and was the focus of substudy 3. The dissemination actions proposed are based on the knowledge and experiences of those working in the cancer control arena with the awareness and understanding of dissemination gaps in their local contexts and the EU.

By using a systems‐oriented approach for data collection (substudy 1), we endeavoured to encourage participants to think beyond one‐off, isolated interventions and instead develop actions that target broader and more sustained systems change. These are places within the dissemination system (i.e. leverage points) where a small shift produces a big change [18]. A systems thinking approach to data analysis [15] for both substudies 1 and 3 categorised almost all actions into the lower two levels of Haynes et al.'s [15] framework of leverage points to change complex systems. Actions at these levels, although highly feasible, are limited in their influence as they target single variables operating mainly in isolation without creating wider systemic impact [31].

Interventions targeting the ‘structures and rules of the system’ utilise rules for accountability or incentives to create change [31]. One way to attain this is by ensuring cancer prevention is on the policy agenda. Three proposed dissemination actions recommended engaging in policy dialogues to align ECAC5 with national plans and electoral manifestos and holding members of parliament accountable through evaluating their commitment to cancer prevention. Cancer prevention has challenges to stand out among other competing priorities, especially as the recent COVID‐19 pandemic has shifted much of the momentum from addressing NCDs to future pandemic preparedness despite the continual rise of NCDs [32, 33].

Advocating for the use of ECAC5 at a policy level, particularly the new recommendations for policymakers included in the 5th edition (Annex S1), may lead to a trickle‐down effect increasing the speed and ease through which change takes place at the lower levels. Governments and national‐level organisations have a responsibility to support and invest in cancer prevention efforts through funding, resilient health systems and accessible and tailored information for healthier populations [7, 34]. For example, through policies that focus on regulation, education and support governments can facilitate environments in which tobacco use is reduced or eliminated thereby supporting behaviour and lifestyle change at an individual level [35].

Despite most proposed actions falling into the lower levels of the framework [15], these actions addressed the barriers identified by participants when disseminating cancer prevention/public health information to the general public in the EU. The medium of dissemination and the content, that is, how the messages are disseminated, were the two most common barriers identified. Conversely, they were also the most emphasised facilitators alongside collaboration and partnerships with key stakeholders and organisations.

Sixteen proposed actions focused on the medium or channel of dissemination. Difficulties in identifying the most appropriate channels, fragmented delivery of information across channels, and the lack of trust in content delivered via social media decreased dissemination effectiveness. Participants recommended utilising varied channels, most of which were digital (e.g. podcasts, social media), alongside other more traditional mediums such as film, street art and billboards, gamification and promotion in areas of high foot traffic.

Social media was the most preferred medium to disseminate ECAC5. Social media is regarded for its ability to increase the accessibility of health information to minority and lower socioeconomic groups [36] as audience engagement and retention are relatively high [37]. However, for young participants (substudy 3), social media was a double‐edged sword–easily accessible to a vast and diverse audience, but difficult to sift through and promote accurate and trustworthy information. Misinformation related to smoking, vaccines, especially the human papilloma virus vaccine, and NCDs is especially high [38]. Platforms like youtube have a significant amount of misleading information, primarily anecdotal, with a high probability of being found by the lay audience [10].

Misinformation can be addressed when the facts are delivered by news organisations and by experts debunking false claims [39]. However, although *interviews with ECAC5 scientific experts on the evidence behind the recommendations* were rated as the most feasible dissemination action, it was not considered the most impactful. Participants perceived public figures, celebrities and influencers to be more effective disseminators, especially when considered trustworthy and relevant in their respective contexts. The Finnish government utilised social media influencers for COVID‐19 campaigns to use their own communication style to successfully influence public attitudes and social norms [40].

Alongside partnerships with the aforementioned stakeholders, interestingly, both substudies 1 and 3 recommended involving nontypical partners such as insurance and tourism companies to offer discounts and benefits for healthy lifestyle behaviours. These partners, also known as knowledge brokers, facilitate the transfer of public health information to intended users across various audiences and sectors [41]. Partnerships with people and organisations were identified as an important facilitator and hence, were featured in twelve proposed dissemination actions.

Content was a key obstacle when disseminating to the general public, particularly when technical/medical or stigmatising terms were used. Cancer‐related content is often viewed through a negative lens, causing feelings of fear and overwhelm [42]. Attributes such as morality, deviance and shame are especially salient for cancers driven primarily by individual behaviour [43, 44]. According to the Intertwined Process Model, when the message being communicated poses a threat to individual freedom, it results in more negative attitudes and decreases the likelihood of undertaking the promoted behaviour [45]. This can be used as a lens from which the public may view the ECAC5. Another hurdle is the limited relevance of cancer prevention to younger audiences and those who have not encountered this chronic disease. Invincible thinking, prevalent in young adults, increases risk‐taking behaviours due to the perception that it will not affect them [46, 47].

To address these challenges and increase the palatability of the ECAC5, four of the proposed dissemination actions encouraged using strengths‐based language, humour, simple but engaging graphics and leveraging social media trends. While an emphasis on content primarily reflects a communication approach, diversifying how the ECAC5 is communicated enhances its likelihood of being applicable to a broader audience and hence warrants consideration when aiming to strengthen dissemination efforts.

Although participants did not focus in detail on tailoring content to various target groups, a few (*n* = 4) strategies suggested targeting content and dissemination based on individual cancer risk or demographics like age and socioeconomic status. Culture‐specific information from trustworthy sources across a broad variety of channels has been identified to overcome barriers when disseminating to a diverse audience [48]. Customising content to the intended audience successfully influenced the decision‐making practices of health policy‐makers [49], supporting the recommendation by substudy 1 participants to use policy briefs when disseminating ECAC5 to policymakers. Although sharing personal stories was identified as a facilitator by substudy 3 participants, as it may combat ‘invincible thinking’ and increase the salience of health messages in younger audiences [46], it did not translate into a proposed action.

Participants in substudy 3 identified gaps in the knowledge of some cancer risk factors over others likely due to their unbalanced promotion, consequently resulting in the ineffective adoption of the ECAC. European research shows that public awareness of risk factors like tobacco is easier to identify in comparison to others such as alcohol, overweight, breastfeeding and infections [50, 51]. Ritchie and colleagues [6] found that some health promoters did not disseminate ECAC4 in its entirety, potentially influencing the awareness of some recommendations over others. Substudy 1 participants recommended the provision of a toolkit of how to disseminate the ECAC5 which can avoid its biased promotion, while substudy 3 participants planned pilot projects on how to disseminate ECAC in its entirety.

A multipronged approach involving the simultaneous dissemination of ECAC5 across several channels was another suggested action (substudy 1). McCormack et al. [52] found that a combination of various communication formats and multicomponent dissemination strategies led to better uptake of health‐related evidence than singular interventions. Similarly, Chapman et al. [36] suggest a combination of dissemination strategies, delivered frequently and intensely over time to maximise the potential for behavioural change. Repeated information from a variety of sources also increases trust [53].

Yet, increasing the reach of the ECAC5 does not guarantee its adoption. What is necessary is to increase people's capability, opportunity and motivation to apply the ECAC5 to reduce their cancer risk and improve their health [54]. Although health literacy was identified as a barrier by several participants (substudy 2), this did not feature in any proposed dissemination action. Low levels of health and digital literacy have been linked to increased misconceptions about cancer, reduced information‐seeking [55], as well as a reduced likelihood of using trusted sources for information [56]. Therefore, actions that improve the health literacy of the EU general public (i.e. at the ‘goals’ level [15]) might incidentally increase the reach of the ECAC5. Interventions at the ‘paradigm’ level [15] target population‐level changes in values and beliefs [31]. In the context of ECAC5, it could mean an increased and sustained public appetite for cancer prevention information with the belief that it can reduce personal cancer risk. Such beliefs are precursors to taking individual action in the form of behavioural changes outlined in the ECAC5.

Haynes et al. [15] state that change occurring at the ‘paradigm’ and ‘goals’ level is harder to achieve but more likely to be transformative and long term. Systems change requires action at multiple levels of this hierarchical framework [15], and although most of the proposed actions were categorised at the lower levels, if successful, over time this can create a shift in perspective and towards achieving change at the upper levels. For example, we hypothesise that the adoption of the ECAC5 by individuals (through dissemination actions at the lower level) will change attitudes and behaviours towards personal cancer risk reduction. As the ECAC5 reaches more people, over time, this will lead to a cumulative shift in societal norms, values and beliefs (paradigm level) to prioritise cancer prevention and control.

### 4.1 Strengths and limitations

Developing dissemination actions begins with a deep consideration of the needs and perspectives of its beneficiaries [26]. Acknowledging the barriers and facilitators when disseminating evidence‐based health information to the EU general public was an antecedent to the identification of dissemination actions in substudy 3. Although such conversations occurred incidentally in substudy 1, and therefore not captured, we compensated by requesting substudy 2 participants for the same. Similar themes emerged across all three groups, strengthening the results. These data were useful in understanding the current dissemination landscape while enabling us to evaluate the relevance of the proposed dissemination actions for ECAC5. Future studies of a similar nature would benefit from employing more visual analysis (e.g. causal loop diagramming) to explore barriers and facilitators of dissemination. This may support problem understanding thereby leading to better identification of solutions [15], and subsequently the identification of more innovative and impactful dissemination actions.

As we employed purposeful sampling for all three substudies, there is potential for selection bias. Due to the idiosyncrasy of ECAC5 as a tool targeting to the commonalities across the EU and not national specificities, our aim was to select participants from EU stakeholders engaged in cancer prevention/public health dissemination activities. These EU‐level organisations, with have a broad understanding of an overall EU context, usually target the EU general public as a whole, or provide coordinated strategies across their national member organisations. We acknowledge the diverse and contextually specific nature of each EU country, which may result in differences in the applicability of some dissemination actions. However, it was difficult to identify country‐specific stakeholders to participate. Future research could use the proposed actions as a stepping stone to adapt, test and implement more country‐specific dissemination activities.

To build on and strengthen workshop outcomes from substudy 1, we attempted to consult a larger pool of stakeholders via an online survey (substudy 2). Although there was a concentrated effort to increase the response rate through snowball sampling and promotion within our networks, we were unable to control the extent of the survey's reach. However, sufficient data were obtained for this small substudy, as reflected in the similar feasibility and impact scores between participants in substudies 1 and 2, and the consistent identification of barriers and facilitators across participants in substudy 2. The inability to gather feasibility and impact scores in substudy 3, due to time constraints, was also a limitation resulting in the inability to provide strong commentary on this data. To meaningfully engage stakeholders and honour their contributions, it is essential to overcome practical constraints such as limited time, resources and the capacity to offer gestures of appreciation.

For most participants, this was their first time engaging with systems thinking and design thinking approaches. Limited understanding, coupled with time and resource limitations, resulted in adaptation of activities. Without visualising the dissemination system, leverage points, especially higher up in the framework hierarchy, are harder to isolate and to identify. This possibly led to no actions being proposed at the two uppermost levels of Haynes et al.'s [15] framework: ‘paradigm’ and ‘goals’, which are most impactful at transforming system behaviour and outcomes [31]. Due to this, we forfeited the use of a systems thinking approach for the workshop with younger stakeholders (substudy 3) and instead opted to use design thinking which shares similarities with systems thinking. Design thinking proved to be an engaging process to critically thinking about dissemination and successfully led to the identification of four projects for the ECL Youth Ambassadors to test before the launch of the ECAC5.

## 5 Conclusion

Using novel methods (systems and design thinking) in three stakeholders' substudies, we have identified a set of key actions that participants evaluated as feasible and impactful, to successfully disseminate ECAC5. This broad range of 40 actions enables EU countries to choose those that are most suitable to their unique populations and contexts. These strategies are not only useful to further the promotion of ECAC5 and cancer prevention information but may be transferable to support increasing the awareness of other NCDs. These studies also aimed to foster collaboration with EU stakeholders, by engaging them in collective participatory activities. We expect that the upcoming dissemination of the ECAC5 through the proposed actions could incrementally contribute to shifts towards the upper levels of Haynes et al., [15] framework over time by ensuring that cancer prevention information (ECAC5) is more accessible (medium), digestible (content) and supported by trusted stakeholders (partnerships).

## Conflict of interest

The authors declare no conflict of interest. Where authors are identified as personnel of the International Agency for Research on Cancer/World Health Organization, the authors alone are responsible for the views expressed in this article and they do not necessarily represent the decisions, policy or views of the International Agency for Research on Cancer/World Health Organisation.

## Author contributions

EDS was responsible for data collection, data analysis and writing the first version of the manuscript. CE and DR were responsible for data collection. HZ and JS gave critical revisions on the intellectual content of the manuscript. All authors approved the final manuscript.

## Supporting information


**Annex S1.** European Code Against Cancer, 5th edition. © 2026 International Agency for Research on Cancer / WHO. Used with permission.

**Annex S2.** Online survey with EU stakeholders involved in cancer prevention dissemination.

## Data Availability

The data that support the findings of this study are available from the corresponding author upon reasonable request. The data are not publicly available due to privacy or ethical restrictions.
