# Supplementary material for: Using systems thinking to guide the dissemination of the European code against cancer, 5th edition
Source: Mol Oncol. 2026 Jan 16;20(1):170–87. doi: 10.1002/1878-0261.70195 (PMC12809476; doi:10.1002/1878-0261.70195)
Supplement: Supplementary file 2 — Annex S2. Online survey with EU stakeholders involved in cancer prevention dissemination. [file MOL2-20-170-s001.pdf]

Online Survey with EU Stakeholders involved in cancer prevention dissemination

Thank you for considering taking part in this survey.

Our aim is to gather and learn from the **knowledge of experts** from cancer control and/or public health organisations across the EU to inform us **on the most effective strategies for the dissemination of cancer prevention information**. This will inform our efforts to identify and improve the awareness of the upcoming European Code Against Cancer, 5th Edition (ECAC5).

This short questionnaire (10 minutes) is divided into four parts.
Section 1: Barriers and Facilitators when disseminating to the General Public in the EU
Section 2: Evaluating dissemination strategies for the upcoming ECAC5
Section 3: Demographics

Once again, we appreciate you taking the time to share your knowledge and we value your input.

When you submit this form, it will not automatically collect your details like name and email address unless you provide it yourself.

Barriers and Facilitators experienced when disseminating to the General Public

In a few words, what are some of the key barriers and facilitators you have experienced when engaging in dissemination activities to the general public

1. **Barriers** experienced when engaging in cancer prevention and/or public health dissemination activities (e.g., health promotion/education, information sharing) to the **general public**

Enter your answer

2. **Factors that enhance** effective dissemination when engaging in cancer prevention and/or public health dissemination activities (e.g., health promotion/education, information sharing) to the **general public**

Enter your answer

Next

Evaluating dissemination strategies for the ECAC5

We have conducted a pilot with a small group of stakeholders from EU-Level cancer control/public health organisations to brainstorm possible dissemination strategies for the upcoming European Code Against Cancer, 5th Edition (ECAC5)

Please go through the list below and rate each proposed strategy for its:
- **feasibility** (i.e., are these dissemination strategies possible and easy to implement) and
- **potential impact** (i.e., are they effective, in that, they will reach the general population in the EU and in turn improve cancer prevention).

This will help us evaluate and ascertain their value for the dissemination of ECAC5.

Please note that this list is in random order.

3. Engage public figures and celebrities (e.g., football players, national champions who were patients themselves) to consistently promote the message of cancer prevention and the ECAC5. *

	Not at all	A little	Neutral	Fairly	Very
Feasible	○	○	○	○	○
Impacful	○	○	○	○	○

4. Partner with non-traditional stakeholders, and private or public organisations outside of cancer prevention and public health. Take care to ensure no conflict of interests (i.e., commercial determinants of health)

   For example: banks, telecoms, insurance companies, sports organisations. *

	Not at all	A little	Neutral	Fairly	Very	No
Feasible	○	○	○	○	○	○
Impacful	○	○	○	○	○	○

5. Promote ECAC5 in physical formats in high visibility settings

For example: Family doctor/general practice and hospital waiting rooms, pharmacies, etc. *

	Not at all	A little	Neutral	Fairly	Very
Feasible	◯	◯	◯	◯	◯
Impacful	◯	◯	◯	◯	◯

6. Make ECAC5 available in an online version with clear and simple graphics that can be printed and easily translated *

	Not at all	A little	Neutral	Fairly	Very
Feasible	◯	◯	◯	◯	◯
Impacful	◯	◯	◯	◯	◯

7. Create an ECAC5 communication toolkit (i.e., press packs), including press releases for mass media. *

	Not at all	A little	Neutral	Fairly	Very
Feasible	◯	◯	◯	◯	◯
Impacful	◯	◯	◯	◯	◯

8. Leverage scientific experts involved in ECAC5 for interviews to explain more about the evidence behind the recommendations of the European Code *

	Not at all	A little	Neutral	Fairly	Very
Feasible	◯	◯	◯	◯	◯
Impacful	◯	◯	◯	◯	◯

9. Purchase targeted advertisements on social media so the ECAC5 can reach a wide but targeted cross-section of the public *

	Not at all	A little	Neutral	Fairly	Very
Feasible	◯	◯	◯	◯	◯
Impacful	◯	◯	◯	◯	◯

10. Work with educators and teachers to promote the ECAC5 to children and adolescents.

For example: In educational settings such as schools, youth centres *

	Not at all	A little	Neutral	Fairly	Very
Feasible	◯	◯	◯	◯	◯
Impacful	◯	◯	◯	◯	◯

11. Garner support within each country by forming alliances with national-level patient, scientific and medical associations for dissemination and advocacy with decision-makers *

	Not at all	A little	Neutral	Fairly	Very
Feasible	◯	◯	◯	◯	◯
Impacful	◯	◯	◯	◯	◯

12. Use social media platforms effectively, and create content on each recommendation of the ECAC5.

For example: Instagram Reels (short videos that hold the audience's attention) *

	Not at all	A little	Neutral	Fairly	Very
Feasible	◯	◯	◯	◯	◯
Impacful	◯	◯	◯	◯	◯

13. Create targeted dissemination formats and channels to subgroups in the general public (e.g., by age, gender, vulnerable or at-risk populations) to improve engagement with ECAC5 *

	Not at all	A little	Neutral	Fairly	Very
Feasible	◯	◯	◯	◯	◯
Impacful	◯	◯	◯	◯	◯

14. Engage children and youth in competitions and gamification to promote ECAC5 *

	Not at all	A little	Neutral	Fairly	Very
Feasible	◯	◯	◯	◯	◯
Impacful	◯	◯	◯	◯	◯

15. Push for the use of the ECAC5 at policy level and align with national plans in place by engaging in policy dialogues to put cancer prevention on the agenda. *

	Not at all	A little	Neutral	Fairly	Very
Feasible	◯	◯	◯	◯	◯
Impacful	◯	◯	◯	◯	◯

16. Provide tailored information to explain ECAC5 to policymakers.

For example: Policy briefs and other formats suited to communicating with policymakers *

	Not at all	A little	Neutral	Fairly	Very
Feasible	◯	◯	◯	◯	◯
Impacful	◯	◯	◯	◯	◯

17. Facilitate coalitions of like-minded organisations to promote the ECAC5 at political level nationally *

	Not at all	A little	Neutral	Fairly	Very
Feasible	○	○	○	○	○
Impacful	○	○	○	○	○

18. Advocate for incorporating the implementation of ECAC5 at national-level electoral manifestos *

	Not at all	A little	Neutral	Fairly	Very
Feasible	○	○	○	○	○
Impacful	○	○	○	○	○

19. Host an annual EU-level ECAC forum to show examples of promotion, describe impact and provide the opportunity to showcase and discuss experiences of utilising and implementing ECAC5 *

	Not at all	A little	Neutral	Fairly	Very
Feasible	○	○	○	○	○
Impacful	○	○	○	○	○

20. Join forces and partner with other European projects/initiatives with common values (e.g., equity, human rights) to amplify ECAC5 recommendations – making use of their existing audience *

	Not at all	A little	Neutral	Fairly	Very
Feasible	○	○	○	○	○
Impacful	○	○	○	○	○

21. Co-create ECAC5 dissemination actions and their implementation with key stakeholders *

	Not at all	A little	Neutral	Fairly	Very
Feasible	○	○	○	○	○
Impacful	○	○	○	○	○

22. Engage with trusted social media influencers specific to each demographic or region/country *

	Not at all	A little	Neutral	Fairly	Very
Feasible	○	○	○	○	○
Impacful	○	○	○	○	○

23. Create strengths-based and positive promotion of the ECAC5 (e.g., with humour) to increase motivation to engage with it, while highlighting how it can be a tool (solution) to prevent cancer. *

	Not at all	A little	Neutral	Fairly	Very
Feasible	○	○	○	○	○
Impacful	○	○	○	○	○

24. Learning from comparable and successful dissemination campaigns to extrapolate best practices

For example: Campaigns on HIV prevention and HPV vaccination *

	Not at all	A little	Neutral	Fairly	Very
Feasible	○	○	○	○	○
Impacful	○	○	○	○	○

25. Provide a toolkit (best practice examples, guidance) of dissemination strategies that are translatable across a variety of contexts (e.g., applicable to various countries in the EU) *

	Not at all	A little	Neutral	Fairly	Very
Feasible	◯	◯	◯	◯	◯
Impacful	◯	◯	◯	◯	◯

26. Identify and use creative and varied channels of dissemination, ensuring the right channels of transmission for the intended audience.

For example: Podcast interviews with patient representatives and advocates; *YouTube* channel on cancer prevention topics; storytelling through films; cartoons; short videos) *

	Not at all	A little	Neutral	Fairly	Very
Feasible	◯	◯	◯	◯	◯
Impacful	◯	◯	◯	◯	◯

27. Are there any other dissemination actions (not covered in the list above) that you would like to identify and tell us about?

Enter your answer

Back Next

Demographics

Once again, this is an anonymous survey. However, we are requesting information on the type of organisation you are employed at and your employment role to better inform us in who contributed to this survey.

The data from this section will be aggregated if/when used.

28. Please choose the most appropriate option to describe your organisation *

- [ ] Cancer Prevention
- [ ] Cancer Treatment
- [ ] Cancer Research / Epidemiology
- [ ] Public Health Organisation
- [ ] Civil Society / Non-Governmental Organisation
- [ ] Advocacy Group
- [ ] Other

29. What is your role in the aforementioned organisation? (E.g., advocacy expert, communication specialist, etc.) *

Enter your answer

30. We hope to continue our dissemination consultation at the upcoming European Public Health Conference in Lisbon on 12 November, 2024.

We are organising an in-person participatory consultation workshop. More details here: https://ephconference.eu/2024-pre-conference-programme-640

If you are planning on attending, and would like to participate please email **dsouzae@iarc.who.int** to highlight your interest and so that we may keep you informed.

Back **Submit**
